# Myeloid cell leukemia-1 dependence in acute myeloid leukemia: a novel approach to patient therapy

**DOI:** 10.18632/oncotarget.26579

**Published:** 2019-02-08

**Authors:** Tapan Mahendra Kadia, Hagop M. Kantarjian, Marina Konopleva

**Affiliations:** ^1^ The University of Texas MD Anderson Cancer Center, Houston, Texas, USA

**Keywords:** AML, BCL-2, MCL-1, flavopiridol, alvocidib

## Abstract

Acute myeloid leukemia (AML) is the most common form of acute leukemia in adults, affecting approximately 21,000 people annually (nearly 11,000 deaths) in the United States. B-cell lymphoma 2 (BCL-2) family proteins, notably myeloid cell leukemia-1 (MCL-1), have been associated with both the development and persistence of AML. MCL-1 is one of the predominant BCL-2 family members expressed in samples from patients with untreated AML. MCL-1 is a critical cell survival factor for cancer and contributes to chemotherapy resistance by directly affecting cell death pathways. Here, we review the role of MCL-1 in AML and the mechanisms by which the potent cyclin-dependent kinase 9 inhibitor alvocidib, through regulation of MCL-1, may serve as a rational therapeutic approach against the disease.

## INTRODUCTION

Acute myeloid leukemia (AML) is the most common form of acute leukemia in adults. According to the American Cancer Society, the incidence of AML has increased 1.7% per year from 2004 to 2013 [1]. In 2017 in the United States, it was estimated that AML affected approximately 21,000 people and resulted in nearly 11,000 deaths. Moreover, although death rates from other acute and chronic leukemias decreased by about 1% per year from 2005 to 2014, mortality rates remained consistent for AML [1]. AML is characterized by the infiltration of bone marrow, blood, and other tissues by poorly differentiated hematopoietic cells. It displays distinct clinicopathologic, cytogenetic, and genetic characteristics; these and other factors are used to categorize patients according to most appropriate treatment, prognosis, risk of resistance, or potential for treatment-related mortality [2–4].

Alterations in apoptotic pathways are common in human malignancies and, in certain cancers, essential for tumorigenesis and cancer maintenance [5]. In this context, B-cell lymphoma 2 (BCL-2) family proteins, notably myeloid cell leukemia-1 (MCL-1), are known for their role in both the development and persistence of AML [6, 7], and are often associated with cancer-cell survival and resistance to chemotherapy [8, 9]. Furthermore, as explored in detail in this article, gene expression analysis suggests that MCL-1 is one of the predominant BCL-2 family members expressed in samples from patients with untreated AML [10]. Here, we review the role of MCL-1 in AML and the mechanisms by which the potent cyclin-dependent kinase (CDK) 9 inhibitor alvocidib may serve, through regulation of MCL-1, as a rational therapeutic approach against the disease.

## CURRENT AND NOVEL TARGETED THERAPIES FOR ACUTE AND CHRONIC LEUKEMIAS

Standard treatment for AML consists of intensive induction chemotherapy, followed by consolidation chemotherapy or allogeneic hematopoietic stem cell transplantation (HSCT) in patients with high-risk disease. Select groups of patients are offered lower-intensity therapy (eg, hypomethylating agents) or investigational therapy in a clinical trial [11]. Patients with AML have a poor prognosis, particularly older patients [1, 12–18]. Complete responses are achieved in only approximately 40% of patients aged 70 years or older compared with approximately 70% of patients aged 60 years or younger [19]. Despite marked advances in understanding the biology and genetics of AML, therapeutic progress has been limited [1]. For the past 30 years, cytarabine and anthracycline-based induction chemotherapy has been the standard of care [20–23].

In recent years, multiple efforts have focused on the development of targeted therapeutics directed against relevant proteins (Table 1) [24–61]. These include approved agents such as the kinase inhibitor midostaurin for patients with FLT3 mutation-positive AML (Rydapt^®^, Novartis Pharmacueticals Corporation, East Hanover, NJ, USA); the isocitrate dehydrogenase inhibitors (IDH) ivosidenib (Tibsovo^®^, Agios Pharmaceuticals, Cambridge, MA, USA) and enasidenib (Idhifa^®^, Celgene Corporation, Summit, NJ, USA and Agios Pharmaceuticals, Cambridge, MA, USA) for patients with IDH1 mutation-positive and IDH2 mutation-positive AML, respectively; and the CD33-targeted antibody-drug conjungate gemtuzumab (Mylotarg™, Wyeth Pharmaceuticals, Philadelphia, PA, USA). Other agents under investigation in AML [62] include poly ADP-ribose polymerase inhibitors that may trigger irreparable DNA damage [63, 64]; epigenetic drugs that may modulate methylation, acetylation, and cell signaling and cycling [65]; and Aurora kinase inhibitors, which target the Aurora family of serine/threonine kinases, enzymes essential for multiple processes during mitosis, including chromosome alignment, centrosomal maturation, mitotic spindle formation, and cytokinesis [66–69]. Additional compounds have received orphan drug designation for the treatment of AML [70], including the histone deacetylase inhibitor pracinostat (MEI Pharma, San Diego, CA, USA) and the CDK9 inhibitor alvocidib (Tolero Pharmaceuticals, Lehi, UT, USA).

**Table 1:** Targeted-therapies for AML molecules^*^

**FMS-like tyrosine kinase 3 kinase inhibitors** Sorafenib (Nexavar^®^; Bayer Healthcare Pharmaceuticals Inc, Whippany, NJ, USA) [24–28] Midostaurin (Rydapt^®^; Novartis, East Hanover, NJ, USA) [29–31] Quizartinib (Daiichi Sankyo Group, Parsippany, NJ, USA) [32, 33] Crenolanib besylate (Arog Pharmaceuticals, Inc, Dallas, TX, USA) [34, 35] Gilteritinib (Astellas Pharma, Tokyo, Japan) [36]
**Antibody-based therapies** Gemtuzumab ozogamicin (anti-CD33; Mylotarg™, Pfizer, New York, NY, USA) [37–39] IMGN779 (anti-CD33; ImmunoGen, Waltham MA, USA) [40] MCLA117 (bispecific anti-CLEC12A×CD3; Merus, Cambridge, MA, USA) [41] CAR T cells [42] Flotetuzumab (bispecific anti-CD123×CD3; MacroGenics, Rockville, MD, USA) [43] IMGN632 (anti-CD123; ImmunoGen, Waltham MA, USA) [44]
**Isocitrate dehydrogenase 1 and 2** Enasidenib (Idhifa^®^; Celgene Corporation, Summit, NJ, USA and Agios Pharmaceuticals, Cambridge, MA, USA) [45] Ivosidenib (Tibsovo^®^; Agios Pharmaceuticals, Cambridge, MA, USA) [46]
**Other small molecule compounds** HDAC inhibitors: – vorinostat (Zolinza^®^; Merck, Kenilworth, NJ, USA) [47–50] – panobinostat (Farydak^®^; Novartis, Cambridge, MA, USA) [51, 52] – romidepsin (Istodax^®^; Celgene Corporations, Summit, NJ, USA) [53–55] – SB939 (Pracinostat^®^; MEI Pharma, San Diego, CA, USA) [56] – SNDX 275 (Entinostat^®^; Syndax, Waltham, MA, USA) [57] BCL-2 inhibitor: venetoclax (Venclexta^®^; AbbVie-Genentech, North Chicago, IL, USA) [58–61]

^*^Not inclusive of all compounds that have been or are undergoing clinical study.

Abbreviations: AML, acute myeloid leukemia; BCL-2, B-cell lymphoma 2; CAR, chimeric antigen receptor; HDAC, histone deacetylase.

Alvocidib (also known as L86-8275, NSC 649890, and flavopiridol) is a semisynthetic flavonoid derived from rohitukine, an alkaloid isolated from a plant indigenous to India. Early studies described it as a potent compound capable of reversibly blocking cell progression at more than one point of the cell cycle, as addressed in this review [71].

## CLINICAL STUDIES WITH ALVOCIDIB

Alvocidib was the first CDK inhibitor to enter human clinical trials [72]. Multiple preclinical [6, 73–77] and clinical [49, 78–85] studies have been conducted, leading to combination therapies that, based on the mechanism(s) of action of alvocidib, have shown clinical activity supporting futher development. These include the addition of alvocidib to regimens containing cytarabine and mitoxantrone (ACM [formerly FLAM]) [81, 82, 84] and fludarabine/rituximab, investigated in a phase I trial in patients with mantle cell lymphoma, chronic lymphocytic leukemia (CLL), or indolent B-cell non-Hodgkin's lymphoma [83]. In fact, in earlier phase II trials, the ACM regimen showed an overall complete remission rate of 67-80% in patients with newly diagnosed, poor-risk AML, with low rates of morbidity and mortality [81, 86, 87]. More recently, these observations were extended to a multicenter randomized phase II trial in adults with newly diagnosed AML with intermediate- and adverse-risk cytogenetics [84]. ACM led to higher complete remission rates than 7+3 (70% vs 46%; *p* = 0.003); this improvement persisted after 7+3 ± 5+2 (70% vs 57%; *p* = 0.08), further illustrating the efficacy of ACM induction in patients with newly diagnosed AML [84, 85]. Importantly, ACM was not associated with increased toxicity relative to 7+3, with similar rates of tumor lysis syndrome (TLS; 8% vs 7%, respectively). However, two ACM-treated patients compared with one 7+3-treated patient experienced early death due to TLS, and three grade 4 TLS toxicities were reported, all in patients treated with ACM [84].

Combination therapy with other targeted agents has also been studied. In a phase I trial, alvocidib was investigated in combination with the histone deacetylase inhibitor vorinostat in patients with relapsed, refractory, or poor prognosis acute leukemia or refractory anemia with excess type-2 blasts [49]. In this study, no objective responses were achieved, although 13 of 26 evaluable patients exhibited stable disease. The combination of alvocidib with vorinostat was well tolerated, with fatigue being the most common non-hematologic adverse event. No patient experienced TLS, but this study was designed to monitor and prophylactically treat TLS [49]. Alvocidib was also studied in combination with the proteasome inhibitor bortezomib in a phase I trial of patients with recurrent or refractory B-cell neoplasms [80] and as a bolus infusion in a similar patient population [79]. These studies showed that the regimen was clinically active in these patients and, importantly, the nonhybrid schedule regimen was recommended for subsequent studies [79, 80]. Based on preclinical findings that alvocidib potentiated imatinib-mediated cell death in human Bcr-Abl+ cells, a phase I trial of alvocidib plus imatinib in advanced Bcr-Abl+ leukemias was initiated [78]. These studies, along with others, led to the designation of alvocidib as an orphan drug in 2014 [70].

## ALVOCIDIB AND CYCLIN-DEPENDENT KINASES: EFFECTS ON CELL CYCLE AND GENE EXPRESSION

One of the most relevant hallmarks of cancer cells is their ability to maintain proliferation, an effect directly associated with a deregulated cell cycle [5, 88]. Unconstrained proliferation secondary to the loss of cell-cycle regulation plays a key role in the initiation and progression of cancer. Early studies conducted to identify the mechanism(s) of action of alvocidib showed its inhibitory effects on cell-cycle progression [71, 89–91].

**Figure 1:**
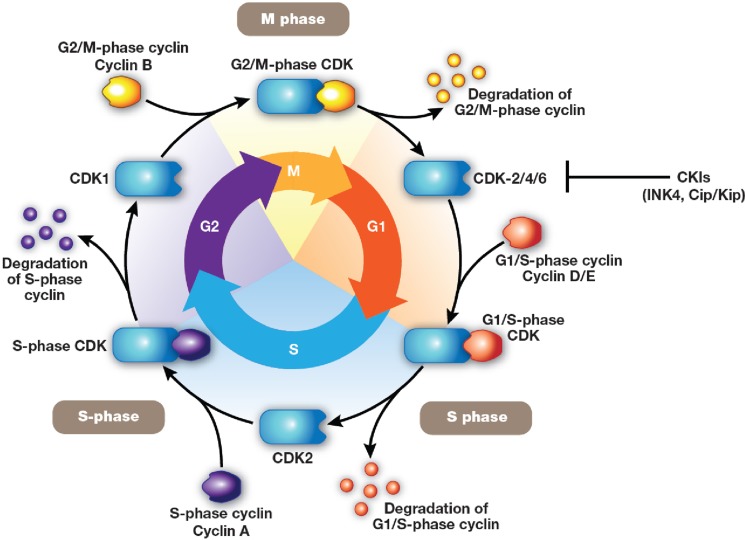
Cell cycle Cells divide and replicate following a precise and strictly regulated process. Cell-cycle progression is coordinated by the activation and degradation of heterodimeric protein complexes formed by catalytic serine/threonine cyclin-dependent kinases (CDK; CDK2/4/6), and their regulatory counterparts (D-type cyclins D1, D2, D3; E-type cyclins E1 and E2; A-type cyclins A1 and A2). [95–97] The activity of CDK/cyclin complexes is negatively regulated by two families of CDK inhibitors: INK4 (p16Ink4a, p15Ink4b, p18Ink4c, p19Ink4d, which inhibit the cyclin D-dependent CDK2/4/6) and Cip/Kip (p21waf1, p27kip1, p57kip2, which inhibit CDK2/cyclin E or A) [98, 99].

**Figure 2:**
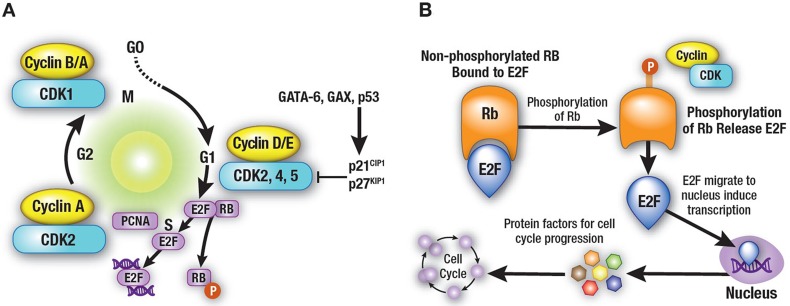
Cell-cycle regulatory proteins **(A)** Cell-cycle regulatory proteins are associated with each other through the retinoblastoma protein (pRb), which is phosphorylated by the activated cyclin D–CDK4/6 complexes, a process that regulates pRb-modulated availability of the transcription factor E2F. **(B)** Unphosphorylated pRb blocks the availability of E2F, while cyclin D–CDK4/6-mediated pRb phosphorylation releases E2F, triggering the transcription of early E2F responsive genes, including cyclins E and A. [100] The effects of alvocidib on cell-cycle progression are linked to its ability of inhibiting several CDKs including CDK1, 2, and 4/6 [71, 89–91].

Progression through the cell cycle is monitored at cell-cycle checkpoints where potential defects in DNA synthesis and/or chromosome segregation are regulated through checkpoint activation and cell-cycle arrest [92, 93]. This regulatory process involves multiple proteins, including cyclins, CDKs, and CDK inhibitors (CKIs), leading ultimately to CDK inhibition [94]. Mutations in CDKs and their regulators (cyclins and CKIs), as well as epigenetic repression of these genes, have been shown to be directly associated with deregulation of the cell cycle in multiple types of cancers [95, 96]. Through the cell cycle, cells divide and replicate following a precise and strictly regulated process. This is coordinated by the activation and degradation of heterodimeric protein complexes formed by catalytic serine/threonine CDKs, notably CDK2/4/6, and their regulatory counterparts, a subset of cyclins directly involved in driving the cell cycle. Regulatory cyclins include D-type cyclins (D1, D2, and D3), which bind preferentially to CDK4/6, and E-type (E1 and E2) and A-type (A1 and A2) cyclins, which bind to CDK2 [95–97]. CDK/cyclin activity is negatively regulated by two families of CKIs: the INK4 (p16Ink4a, p15Ink4b, p18Ink4c, and p19Ink4d, which inhibit the cyclin D-dependent CDK2/4/6) and Cip/Kip (p21waf1, p27kip1, and p57kip2, which inhibit CDK2/cyclin E or A) (Figure 1) [95, 96]. In addition, cell-cycle regulatory proteins associate with each other through the retinoblastoma protein (pRb), which is phosphorylated by activated cyclin D–CDK4/6 complexes. This process regulates pRb-modulated availability of the transcription factor E2F: unphosphorylated pRb blocks the availability of E2F, while cyclin D–CDK4/6-mediated pRb phosphorylation releases E2F, triggering the transcription of early E2F-responsive genes, including cyclins E and A (Figure 2A–2B) [100]. The effect of alvocidib on cell-cycle progression has been linked to inhibition of several CDKs, including CDK1, 2, and 4/6 [68, 86–88]. The main molecular mechanisms that have been associated with the activity of alvocidib are summarized in Table 2 [49, 71, 73–75, 77, 89, 90, 94, 101–126].

**Table 2:** Main molecular mechanisms involved in the activity of alvocidib

**Modulation of cell cycle** Cell cycle arrest at the G1 phase through inhibition of cell cycle-related CDK1, CDK2, CDK4, and CDK6 [71, 89, 90, 94, 102, 103] CDKI p21^CIP1^ transcriptional inhibition. [49, 73, 103, 104] **Regulation of transcription** Inhibition of non–cell-cycle–related CDK7 and CDK9 [103, 105–107] Potent alteration of the expression of genes involved in cell cycle, cell death, and transcriptional regulation, among others [108–110] **Effects on cell death–apoptosis pathways** Mithocondrial-mediated cell death induction [75, 111] Regulation of MCL-1 [74, 112–114] Regulates the expression of other pro- and anti-apoptotic proteins including BCL-2-[115–121] and IAP-[77, 116, 122–126] family proteins

Abbreviations: BCL-2, B-cell lymphoma 2; IAP, inhibitor of apoptosis proteins; MCL, myeloid cell leukemia.

In addition to its inhibitory effects on cell-cycle–related CDKs, alvocidib exhibits its most potent effects on CDK7 and CDK9, both of which are non–cell-cycle-related and play important roles in transcription and gene expression, including several genes that are critical for cell survival under stress [102, 105–107]. Both CDK7 and CDK9 target the carboxyl-terminal domain (CTD) of RNA polymerase II, controlling, through sequential phosphorylation of different residues, the transcription process at several stages including initiation, elongation, and termination [127, 128]. CDK7 and its associated regulatory proteins cyclin H and MAT1 are part of a complex within transcription factor IIH, which regulates RNA polymerase II during the initiation phase and during promoter clearance [129–131]. CDK9, on the other hand, is part of positive transcription elongation factor b (P-TEFb) and is modulated by its association with cyclins T1, T2a, and T2b, having a unique role in the regulation of RNA polymerase II during productive elongation [132–134].

Targeting gene transcription as a means of controlling the expression of critical proteins has been considered a potentially risky strategy because of nonselective effects that may impact both cancer and normal cells [135, 138]. However, recent studies have shown that a number of highly expressed genes that are either oncogenic (eg, MYC, MYCN, RUNX1) [137–140] and/or provide critical survival advantages (eg, MCL-1) [8] are highly dependent on continuous active transcription. Gene expression relies on *cis*-acting DNA sequences, namely transcription enhancers, which increase transcription independently of their orientation and distance relative to the RNA start site. These transcription enhancers are discrete DNA elements that contain specific sequence motifs with which DNA-binding proteins interact and transmit molecular signals to genes [141, 142]. To maintain adequate levels of short-life proteins such as MCL-1 in cancer cells, the continuous active transcription of the genes coding for these proteins is often driven by large sections of DNA that comprise multiple enhancers, named “super-enhancers” [143–145]. Although the activities of thousands of genes are controlled by enhancer elements, only those genes with especially prominent roles are controlled by super-enhancers [143]. These regions are densely populated by components of the transcription machinery (ie, in addition to transcription factors, co-factors, RNA polymerase II, and CDKs), including targets of alvocidib therapy CDK7, CDK8, CDK9, CDK12, and CDK13 [143, 146, 147]. From a therapeutic standpoint, that most of the genes regulated by “super-enhancers” code for short half-life mRNAs and proteins makes targeting gene transcription a feasible approach in cancer therapy, as highly selective effects may be reached before a global transcriptional down-regulation is achieved [136].

## ALVOCIDIB AND APOPTOSIS IN CANCER CELLS: REGULATION OF MCL-1

Dysregulation and evasion of apoptosis is one of Hanahan and Weinberg's hallmarks of cancer [5, 88]. Cell death by apoptosis may occur through two major signaling pathways: the intrinsic (or mitochondrial) pathway and the extrinsic (or death receptor-mediated) pathway [148, 149]. Early studies provide evidence that exposure of human leukemia cells to alvocidib triggers apoptosis by the mitochondrial rather than the death receptor–mediated pathway [75, 111]. The intrinsic apoptotic pathway, following pro–cell death stimuli, is initiated by mitochondrial outer membrane permeabilization (MOMP) and the release of cytochrome c from the intermembrane space [150]. This process is controlled by the BCL-2 family of proteins [148], which are grouped into three classes: proapoptotic effector proteins, including BAX and BAK, which are responsible for MOMP; anti-apoptotic BCL-2 proteins, such as BCL-2, BCL-xL and MCL-1, which block MOMP; and the BCL-2 homology (BH)3-only proteins BID, BIM, BAD, BIK, HRK, PUMA, BMF, and NOXA, which either activate proapoptotic effectors and/or neutralize anti-apoptotic BCL-2 proteins [148, 151]. Multiple studies have investigated the BCL-2 family of proteins as therapeutic targets. The most advanced of these efforts led to the approval of the BCL-2 inhibitor venetoclax for patients with high-risk CLL [152]. In AML, venetoclax has shown preclinical activity [153], with modest single-agent activity in relapsed/refractory AML [61]. However, preliminary clinical data on venetoclax in combination with DNA methyltransferase inhibitors or low-dose cytarabine have shown encouraging results [58, 59, 60, 154–155]. Venetoclax has shown activity in preclinical studies in acute lymphoblastic leukemia [156, 157] and is currently being investigated in pediatric and adult clinical trials. Because of the risk of TLS, prophylactic use of anti-hyperuricemics and hydration is recommended prior to the first dose of venetoclax [158].

Essential to the mechanism of alvocidib-induced anticancer activity is its effect on the expression of MCL-1, which has been shown to play a critical role in sensitizing cells to cell death [74, 112–114]. High levels of anti-apoptotic MCL-1 and other members of the BCL-2 family (eg, BCL-2, BCL-xL) have been shown to contribute not only to the development of some forms of cancer, but also to providing them with survival advantages and chemotherapy resistance [6–10, 159–161]. In fact, amplification of the MCL-1 gene is one of the most frequent somatic genetic events in human cancer, providing evidence of its central role in the pathogenesis of malignancy [159]. Studies examining the expression of BCL-2 family members, including MCL-1 in primary human hematopoietic subsets and leukemic blasts from patients with AML, have consistently shown high expression levels of MCL-1 transcripts [10]. In a functional *in vivo* study of Myc-induced murine AML with high levels of MCL-1, reduction of MCL-1 levels through haploinsufficiency abrogated AML development, supporting the critical role of MCL-1 in AML pathogenesis [10]. Similar observations in AML have shown that high levels of MCL-1, but not of other anti-apoptotic proteins such as BCL-xL, BCL-2 or BCL-w, are critical to the development and sustained growth of the disease [6]. It is noteworthy that alvocidib has been shown to induce the expression of the anti-apoptotic BCL-2 gene in leukemic blasts in adults with refractory AML [119]. However, expression of BCL-2 does not appear to have a major impact on alvocidib-induced lethality [75, 162]. Furthermore, alvocidib displays synergistic effects when administered with selective BH3 mimetic BCL-2 inhibitors (ABT-199 or venetoclax) in *in vitro* and *in vivo* models of AML [163].

Importantly, the differential effects of MCL-1 versus other apoptosis-related BCL-2 family members may reside in a newly identified fuction of MCL-1. In addition to its role in controlling and opposing cell death, which is related to its localization on the outer mitochondrial membrane, an amino-terminally truncated isoform of MCL-1 was found to be imported into the mitochondrial matrix where it facilitates normal mitochondrial function, membrane potential, ATP production, respiration, cristae ultrastructure, and maintenance of oligomeric ATP synthase [164]. These findings provide key information on how the diverse functions of MCL-1 may contribute to cell homeostasis and function, supporting the evidence that high levels of MCL-1 in human cancers contribute to malignant cell growth and evasion of apoptosis [165]. One distinct difference between MCL-1 and other members of the BCL-2 family is its very short half-life, between 0.5 hours and 4 hours [8, 166], which makes it dependent on continuous and active gene transcription, an effect achieved (as mentioned above) through super-enhancer–driven transcription ultimately modulated by CDK9 [143, 146, 147].

Despite alvocidib showing potent clinical activity against blood cancers, patients develop primary or acquired resistance to treatment throughout their clinical course. To understand the mechanism of acquired resistance to alvocidib in leukemia, an alvocidib-resistant cell line was created in a CLL model [161]. The alvocidib-resistant cell line exhibited high transcriptional activity and increased CDK9 activity to promote RNA polymerase II activity, thereby increasing RNA transcription of alvocidib targets. The alvocidib-resistant cell line also exhibited increased transcription and stability of MCL-1. Of particular importance, knockdown of MCL-1 in the alvocidib-resistant cell line partially restored sensitivity to alvocidib. This suggests that the upregulation and stability of MCL-1, as well as enhanced CDK9 activity, are important components of acquired resistance to alvocidib [161]. The relationship between the level of MCL-1 expression and the response to alvocidib is being examined in a phase II study of patients with AML (NCT02520011).

Other proteins affected by alvocidib and critically involved in the regulation of apoptotic signaling include the inhibitor of apoptosis proteins (IAP) family, a group of eight structurally related proteins with the ability to suppress apoptosis, most notably X-linked IAP (XIAP), c-IAP1, c-IAP2, and survivin [167, 168]. Although c-IAP1 and c-IAP2 exert their inhibitory effects on cell death indirectly by functioning as E3 ubiquitin ligases promoting the ubiquitination of caspase-3 and -7 [169, 170], XIAP binds to and inhibits caspase-3, -7, and -9 [167]. Alvocidib has been shown to down-regulate XIAP at the transcriptional level [77].

## CLINICAL DEVELOPMENT OF MCL-1 SMALL MOLECULE INHIBITORS

Several studies investigating the role that MCL-1 expression plays in cancer development and treatment have resulted in significant efforts to develop compounds, such as alvocidib, that may target this apoptosis-inhibitory protein. Approaches to down-regulate MCL-1 expression have been directed multiple ways: inhibiting its transcription via CDK9 inhibition, as is the case with alvocidib and other CDK inhibitors [113, 114]; at the translational level, as occurring in human leukemia cells exposed to sorafenib [171]; or by targeting protein-protein interactions to directly affect MCL-1 anti-apoptotic activity [8, 172–174].

In addition to alvocidib, there are other MCL-1 inhibitors in development. The small molecule S63845 (Servier Laboratories, Suresnes, France and Novartis, Basel, Switzerland) [174] has been shown to bind with high affinity to the BH3-binding groove of MCL-1, resulting in apoptosis of MCL-1–dependent multiple myeloma, leukemia, and lymphoma cells [174]. In addition, S63845 has been shown to sensitize several solid cancer cell lines (including breast cancer and melanoma) to other therapeutic agents. The small molecule MCL-1 inhibitors AMG 176 (Amgen, Thousand Oaks, CA, USA) [175] and AZD5991 (AstraZeneca, Cambridge, UK) [176] are currently in phase I clinical evaluation (NCT02675452 and NCT03218683, respectively) and continue to accrue patients in the US. Other compounds under clinical study include MIK665 (NCT02992483) and S64315 (NCT02979366). Given the marked interest in MCL-1 inhibitors and the efforts currently underway in academic institutions and pharmaceutical laboratories, a summary of some of these studies has been recently published [172].

## SUMMARY

Since its introduction to the field of cancer therapeutics, alvocidib has received much attention, resulting in an accumulation of knowledge and understanding of the mechanisms affecting cancer cell survival. This has led to the development of promising combination therapies against leukemia, including AML, where efficacious approaches are urgently needed. It is clear that alvocidib, by targeting the CDK9/MCL-1 axis and thereby interfering with one of the main pro-survival proteins, represents a unique compound in an area of research where much effort is being invested. Advances have been made in terms of identifying new strategies and schedules of administration that have greatly improved the clinical activity of alvocidib, notably as part of regimens such as ACM, where results supporting its further development have been recorded, especially in patients with newly diagnosed AML.

## References

[1] American Cancer Society (2017). Cancer Facts & Figures 2017.

[2] Ley TJ, Miller C, Ding L, Raphael BJ, Mungall AJ, Robertson A, Hoadley K, Triche TJ, Laird PW, Baty JD, Fulton LL, Fulton R, Heath SE, Cancer Genome Atlas Research Network (2013). Genomic and epigenomic landscapes of adult de novo acute myeloid leukemia. N Engl J Med.

[3] Grimwade D, Hills RK, Moorman AV, Walker H, Chatters S, Goldstone AH, Wheatley K, Harrison CJ, Burnett AK, National Cancer Research Institute Adult Leukaemia Working Group (2010). Refinement of cytogenetic classification in acute myeloid leukemia: determination of prognostic significance of rare recurring chromosomal abnormalities among 5876 younger adult patients treated in the United Kingdom Medical Research Council trials. Blood.

[4] Grimwade D, Ivey A, Huntly BJ (2016). Molecular landscape of acute myeloid leukemia in younger adults and its clinical relevance. Blood.

[5] Hanahan D, Weinberg RA (2000). The hallmarks of cancer. Cell.

[6] Glaser SP, Lee EF, Trounson E, Bouillet P, Wei A, Fairlie WD, Izon DJ, Zuber J, Rappaport AR, Herold MJ, Alexander WS, Lowe SW, Robb L, Strasser A (2012). Anti-apoptotic Mcl-1 is essential for the development and sustained growth of acute myeloid leukemia. Genes Dev.

[7] Juin P, Geneste O, Gautier F, Depil S, Campone M (2013). Decoding and unlocking the BCL-2 dependency of cancer cells. Nat Rev Cancer.

[8] Wei G, Margolin AA, Haery L, Brown E, Cucolo L, Julian B, Shehata S, Kung AL, Beroukhim R, Golub TR (2012). Chemical genomics identifies small-molecule MCL1 repressors and BCL-xL as a predictor of MCL1 dependency. Cancer Cell.

[9] Wuillème-Toumi S, Robillard N, Gomez P, Moreau P, Le Gouill S, Avet-Loiseau H, Harousseau JL, Amiot M, Bataille R (2005). Mcl-1 is overexpressed in multiple myeloma and associated with relapse and shorter survival. Leukemia.

[10] Xiang Z, Luo H, Payton JE, Cain J, Ley TJ, Opferman JT, Tomasson MH (2010). Mcl1 haploinsufficiency protects mice from Myc-induced acute myeloid leukemia. J Clin Invest.

[11] De Kouchkovsky I, Abdul-Hay M (2016). ‘Acute myeloid leukemia: a comprehensive review and 2016 update’. Blood Cancer J.

[12] Burnett A, Wetzler M, Löwenberg B (2011). Therapeutic advances in acute myeloid leukemia. J Clin Oncol.

[13] Döhner H, Estey E, Grimwade D, Amadori S, Appelbaum FR, Büchner T, Dombret H, Ebert BL, Fenaux P, Larson RA, Levine RL, Lo-Coco F, Naoe T (2017). Diagnosis and management of AML in adults: 2017 ELN recommendations from an international expert panel. Blood.

[14] Döhner H, Estey EH, Amadori S, Appelbaum FR, Büchner T, Burnett AK, Dombret H, Fenaux P, Grimwade D, Larson RA, Lo-Coco F, Naoe T, Niederwieser D, European LeukemiaNet (2010). Diagnosis and management of acute myeloid leukemia in adults: recommendations from an international expert panel, on behalf of the European LeukemiaNet. Blood.

[15] Döhner H, Weisdorf DJ, Bloomfield CD (2015). Acute myeloid leukemia. N Engl J Med.

[16] Kadia TM, Ravandi F, Cortes J, Kantarjian H (2015). Toward individualized therapy in acute myeloid leukemia: a contemporary review. JAMA Oncol.

[17] Kadia TM, Ravandi F, O'Brien S, Cortes J, Kantarjian HM (2015). Progress in acute myeloid leukemia. Clin Lymphoma Myeloma Leuk.

[18] Kantarjian H (2016). Acute myeloid leukemia—major progress over four decades and glimpses into the future. Am J Hematol.

[19] Juliusson G, Antunovic P, Derolf A, Lehmann S, Möllgård L, Stockelberg D, Tidefelt U, Wahlin A, Höglund M (2009). Age and acute myeloid leukemia: real world data on decision to treat and outcomes from the Swedish Acute Leukemia Registry. Blood.

[20] Burnett AK (2012). Treatment of acute myeloid leukemia: are we making progress?. Hematology Am Soc Hematol Educ Program.

[21] Burnett AK, Hills RK, Milligan DW, Goldstone AH, Prentice AG, McMullin MF, Duncombe A, Gibson B, Wheatley K (2010). Attempts to optimize induction and consolidation treatment in acute myeloid leukemia: results of the MRC AML12 trial. J Clin Oncol.

[22] Burnett AK, Russell NH, Hills RK, Hunter AE, Kjeldsen L, Yin J, Gibson BE, Wheatley K, Milligan D (2013). Optimization of chemotherapy for younger patients with acute myeloid leukemia: results of the medical research council AML15 trial. J Clin Oncol.

[23] Dombret H, Gardin C (2016). An update of current treatments for adult acute myeloid leukemia. Blood.

[24] Levis M (2013). FLT3 mutations in acute myeloid leukemia: what is the best approach in 2013?. Hematology Am Soc Hematol Educ Program.

[25] Al-Kali A, Cortes J, Faderl S, Jones D, Abril C, Pierce S, Brandt M, Kantarjian H, Ravandi F (2011). Patterns of molecular response to and relapse after combination of sorafenib, idarubicin, and cytarabine in patients with FLT3 mutant acute myeloid leukemia. Clin Lymphoma Myeloma Leuk.

[26] Konig H, Levis M (2015). Targeting FLT3 to treat leukemia. Expert Opin Ther Targets.

[27] Man CH, Fung TK, Ho C, Han HH, Chow HC, Ma AC, Choi WW, Lok S, Cheung AM, Eaves C, Kwong YL, Leung AY (2012). Sorafenib treatment of FLT3-ITD(+) acute myeloid leukemia: favorable initial outcome and mechanisms of subsequent nonresponsiveness associated with the emergence of a D835 mutation. Blood.

[28] Zhang W, Konopleva M, Shi YX, McQueen T, Harris D, Ling X, Estrov Z, Quintás-Cardama A, Small D, Cortes J, Andreeff M (2008). Mutant FLT3: a direct target of sorafenib in acute myelogenous leukemia. J Natl Cancer Inst.

[29] Cooper BW, Kindwall-Keller TL, Craig MD, Creger RJ, Hamadani M, Tse WW, Lazarus HM (2015). A phase I study of midostaurin and azacitidine in relapsed and elderly AML patients. Clin Lymphoma Myeloma Leuk.

[30] Fischer T, Stone RM, Deangelo DJ, Galinsky I, Estey E, Lanza C, Fox E, Ehninger G, Feldman EJ, Schiller GJ, Klimek VM, Nimer SD, Gilliland DG (2010). Phase IIB trial of oral Midostaurin (PKC412), the FMS-like tyrosine kinase 3 receptor (FLT3) and multi-targeted kinase inhibitor, in patients with acute myeloid leukemia and high-risk myelodysplastic syndrome with either wild-type or mutated FLT3. J Clin Oncol.

[31] Stone RM, DeAngelo DJ, Klimek V, Galinsky I, Estey E, Nimer SD, Grandin W, Lebwohl D, Wang Y, Cohen P, Fox EA, Neuberg D, Clark J (2005). Patients with acute myeloid leukemia and an activating mutation in FLT3 respond to a small-molecule FLT3 tyrosine kinase inhibitor, PKC412. Blood.

[32] Cortes JE, Kantarjian H, Foran JM, Ghirdaladze D, Zodelava M, Borthakur G, Gammon G, Trone D, Armstrong RC, James J, Levis M (2013). Phase I study of quizartinib administered daily to patients with relapsed or refractory acute myeloid leukemia irrespective of FMS-like tyrosine kinase 3-internal tandem duplication status. J Clin Oncol.

[33] Levis M, Ravandi F, Wang ES, Baer MR, Perl A, Coutre S, Erba H, Stuart RK, Baccarani M, Cripe LD, Tallman MS, Meloni G, Godley LA (2011). Results from a randomized trial of salvage chemotherapy followed by lestaurtinib for patients with FLT3 mutant AML in first relapse. Blood.

[34] Randhawa JK, Kantarjian HM, Borthakur G, Thompson PA, Konopleva M, Daver N, Pemmaraju N, Jabbour E, Kadia TM, Estrov Z, Ramachandran A, Paradela J, Andreef M (2014). Results of a phase II study of crenolanib in relapsed/refractory acute myeloid leukemia patients (pts) with activating FLT3 mutations. Blood.

[35] Smith CC, Lasater EA, Lin KC, Wang Q, McCreery MQ, Stewart WK, Damon LE, Perl AE, Jeschke GR, Sugita M, Carroll M, Kogan SC, Kuriyan J, Shah NP (2014). Crenolanib is a selective type I pan-FLT3 inhibitor. Proc Natl Acad Sci U S A.

[36] Perl AE, Altman JK, Cortes J, Smith C, Litzow M, Baer MR, Claxton D, Erba HP, Gill S, Goldberg S, Jurcic JG, Larson RA, Liu C (2017). Selective inhibition of FLT3 by gilteritinib in relapsed or refractory acute myeloid leukaemia: a multicentre, first-in-human, open-label, phase 1-2 study. Lancet Oncol.

[37] Castaigne S, Pautas C, Terré C, Raffoux E, Bordessoule D, Bastie JN, Legrand O, Thomas X, Turlure P, Reman O, de Revel T, Gastaud L, de Gunzburg N, Acute Leukemia French Association (2012). Effect of gemtuzumab ozogamicin on survival of adult patients with de-novo acute myeloid leukaemia (ALFA-0701): a randomised, open-label, phase 3 study. Lancet.

[38] Hills RK, Castaigne S, Appelbaum FR, Delaunay J, Petersdorf S, Othus M, Estey EH, Dombret H, Chevret S, Ifrah N, Cahn JY, Récher C, Chilton L (2014). Addition of gemtuzumab ozogamicin to induction chemotherapy in adult patients with acute myeloid leukaemia: a meta-analysis of individual patient data from randomised controlled trials. Lancet Oncol.

[39] Petersdorf SH, Kopecky KJ, Slovak M, Willman C, Nevill T, Brandwein J, Larson RA, Erba HP, Stiff PJ, Stuart RK, Walter RB, Tallman MS, Stenke L, Appelbaum FR (2013). A phase 3 study of gemtuzumab ozogamicin during induction and postconsolidation therapy in younger patients with acute myeloid leukemia. Blood.

[40] Kovtun Y, Noordhuis P, Whiteman KR, Watkins K, Jones GE, Harvey L, Lai KC, Portwood S, Adams S, Sloss CM, Schuurhuis GJ, Ossenkoppele G, Wang ES, Pinkas J (2018). IMGN779, a Novel CD33-Targeting Antibody-Drug Conjugate with DNA-Alkylating Activity, Exhibits Potent Antitumor Activity in Models of AML. Mol Cancer Ther.

[41] Van Loo PF, Doornbos R, Dolstra H, Shamsili S, Bakker L (2015). Preclinical evaluation of MCLA117, a CLEC12AxCD3 bispecific antibody efficiently targeting a novel leukemic stem cell associated antigen in AML. Blood.

[42] Rafiq S, Purdon TJ, Schultz LM, Brentjens RJ (2016). CD33-directed chimeric antigen receptor (CAR) T cells for the treatment of acute myeloid leukemia (AML). Blood.

[43] Uy GL, Godwin J, Rettig MP, Vey N, Foster M, Arellano ML, Rizzieri DA, Topp MS, Huls G, Lowenberg B, Martinelli G, Paolini S, Ciceri F (2017). Preliminary results of a phase 1 study of flotetuzumab, a CD123 × CD3 bispecific DART® protein, in patients with relapsed/refractory acute myeloid leukemia and myelodysplastic syndrome. Blood.

[44] Kovtun Y, Jones GE, Adams S, Harvey L, Audette CA, Wilhelm A, Bai C, Rui L, Laleau R, Liu F, Ab O, Setiady Y, Yoder NC (2018). A CD123-targeting antibody-drug conjugate, IMGN632, designed to eradicate AML while sparing normal bone marrow cells. Blood Adv.

[45] Ellwood-Yen K, Wang F, Travins J, Chen Y, Yang H, Straley K, Choe S, Dorsch M, Agresta S, Schenkein D, Biller S, Su M (2014). Abstract 3116: AG-221 offers a survival advantage in a primary human IDH2 mutant AML xenograft model. Cancer Res.

[46] Pollyea DA, de Botton S, Fathi AT, Stein EM, Tallman MS, Agresta S, Bowden C, Fan B, Prah M, Yang H, Yen K, Stone RM (2014). 1LBA Clinical safety and activity in a phase I trial of AG-120, a first in class, selective, potent inhibitor of the IDH1-mutant protein, in patients with IDH1 mutant positive advanced hematologic malignancies. Eur J Cancer.

[47] Garcia-Manero G, Othus M, Pagel JM, Radich JP, Fang M, Rizzieri DA, Marcucci G, Strickland SA, Litzow M, Savoie ML, Medeiros BC, Sekeres MA, Lin TL (2016). SWOG S1203: a randomized phase III study of standard cytarabine plus daunorubicin (7+3) therapy versus idarubicin with high dose cytarabine (IA) with or without vorinostat (IA+V) in younger patients with previously untreated Acute Myeloid Leukemia (AML). Blood.

[48] Garcia-Manero G, Tambaro FP, Bekele NB, Yang H, Ravandi F, Jabbour E, Borthakur G, Kadia TM, Konopleva MY, Faderl S, Cortes JE, Brandt M, Hu Y (2012). Phase II trial of vorinostat with idarubicin and cytarabine for patients with newly diagnosed acute myelogenous leukemia or myelodysplastic syndrome. J Clin Oncol.

[49] Holkova B, Supko JG, Ames MM, Reid JM, Shapiro GI, Perkins EB, Ramakrishnan V, Tombes MB, Honeycutt C, McGovern RM, Kmieciak M, Shrader E, Wellons MD (2013). A phase I trial of vorinostat and alvocidib in patients with relapsed, refractory, or poor prognosis acute leukemia, or refractory anemia with excess blasts-2. Clin Cancer Res.

[50] Kirschbaum M, Gojo I, Goldberg SL, Bredeson C, Kujawski LA, Yang A, Marks P, Frankel P, Sun X, Tosolini A, Eid JE, Lubiniecki GM, Issa JP (2014). A phase 1 clinical trial of vorinostat in combination with decitabine in patients with acute myeloid leukaemia or myelodysplastic syndrome. Br J Haematol.

[51] Ocio EM, Herrera P, Olave MT, Castro N, Pérez-Simón JA, Brunet S, Oriol A, Mateo M, Sanz MÁ, López J, Montesinos P, Chillón MC, Prieto-Conde MI, PETHEMA Group (2015). Panobinostat as part of induction and maintenance for elderly patients with newly diagnosed acute myeloid leukemia: phase Ib/II panobidara study. Haematologica.

[52] Tan P, Wei A, Mithraprabhu S, Cummings N, Liu HB, Perugini M, Reed K, Avery S, Patil S, Walker P, Mollee P, Grigg A, D'Andrea R (2014). Dual epigenetic targeting with panobinostat and azacitidine in acute myeloid leukemia and high-risk myelodysplastic syndrome. Blood Cancer J.

[53] Jain N, Odenike O (2010). Emerging role of the histone deacetylase inhibitor romidepsin in hematologic malignancies. Expert Opin Pharmacother.

[54] Klimek VM, Fircanis S, Maslak P, Guernah I, Baum M, Wu N, Panageas K, Wright JJ, Pandolfi PP, Nimer SD (2008). Tolerability, pharmacodynamics, and pharmacokinetics studies of depsipeptide (romidepsin) in patients with acute myelogenous leukemia or advanced myelodysplastic syndromes. Clin Cancer Res.

[55] Odenike OM, Alkan S, Sher D, Godwin JE, Huo D, Brandt SJ, Green M, Xie J, Zhang Y, Vesole DH, Stiff P, Wright J, Larson RA, Stock W (2008). Histone deacetylase inhibitor romidepsin has differential activity in core binding factor acute myeloid leukemia. Clin Cancer Res.

[56] Garcia-Manero G, Montalban-Bravo G, Berdeja JG, Abaza Y, Jabbour E, Essell J, Lyons RM, Ravandi F, Maris M, Heller B, DeZern AE, Babu S, Wright D (2017). Phase 2, randomized, double-blind study of pracinostat in combination with azacitidine in patients with untreated, higher-risk myelodysplastic syndromes. Cancer.

[57] Prebet T, Sun Z, Figueroa ME, Ketterling R, Melnick A, Greenberg PL, Herman J, Juckett M, Smith MR, Malick L, Paietta E, Czader M, Litzow M (2014). Prolonged administration of azacitidine with or without entinostat for myelodysplastic syndrome and acute myeloid leukemia with myelodysplasia-related changes: results of the US Leukemia Intergroup trial E1905. J Clin Oncol.

[58] Lin TL, Strickland SA, Fiedler W, Walter RB, Hou JZ, Roboz GJ, Enjeti A, Fakhoui KM, Darden DE, Dunbar M, Zhu M, Hayslip JW, Wei AH (2016). Phase Ib/2 study of venetoclax with low-dose cytarabine in treatment-naive patients age ≥ 65 with acute myelogenous leukemia. J Clin Oncol.

[59] Pollyea DA, Dinardo CD, Thirman MJ, Letai A, Wei AH, Jonas BA, Arellano ML, Frattini MG, Kantarjian HM, Chyla B, Zhu M, Potluri J, Humerickhouse R (2016). Results of a phase 1b study of venetoclax plus decitabine or azacitidine in untreated acute myeloid leukemia patients ≥ 65 years ineligible for standard induction therapy. J Clin Oncol.

[60] DiNardo CD, Pratz KW, Letai A, Jonas BA, Wei AH, Thirman M, Arellano M, Frattini MG, Kantarjian H, Popovic R, Chyla B, Xu T, Dunbar M (2018). Safety and preliminary efficacy of venetoclax with decitabine or azacitidine in elderly patients with previously untreated acute myeloid leukaemia: a non-randomised, open-label, phase 1b study. Lancet Oncol.

[61] Konopleva M, Pollyea DA, Potluri J, Chyla B, Hogdal L, Busman T, McKeegan E, Salem AH, Zhu M, Ricker JL, Blum W, DiNardo CD, Kadia T (2016). Efficacy and Biological Correlates of Response in a Phase II Study of Venetoclax Monotherapy in Patients with Acute Myelogenous Leukemia. Cancer Discov.

[62] Lancet JE (2013). New agents: great expectations not realized. Best Pract Res Clin Haematol.

[63] Esposito MT, Zhao L, Fung TK, Rane JK, Wilson A, Martin N, Gil J, Leung AY, Ashworth A, So CW (2015). Synthetic lethal targeting of oncogenic transcription factors in acute leukemia by PARP inhibitors. Nat Med.

[64] Wang L, Hamard PJ, Nimer SD (2015). PARP inhibitors: a treatment option for AML?. Nat Med.

[65] Saygin C, Carraway HE (2017). Emerging therapies for acute myeloid leukemia. J Hematol Oncol.

[66] Kelly KR, Freeman CL, Giles FJ, Andreeff M (2015). The clinical development of aurora kinase inhibitors in acute myeloid leukemia. Targeted Therapy of Acute Myeloid Leukemia.

[67] Bavetsias V, Linardopoulos S (2015). Aurora kinase inhibitors: current status and outlook. Front Oncol.

[68] Fathi AT, Wander SA, Blonquist TM, Brunner AM, Amrein PC, Supko J, Hermance NM, Manning AL, Sadrzadeh H, Ballen KK, Attar EC, Graubert TA, Hobbs G (2017). Phase I study of the aurora A kinase inhibitor alisertib with induction chemotherapy in patients with acute myeloid leukemia. Haematologica.

[69] Goldberg SL, Fenaux P, Craig MD, Gyan E, Lister J, Kassis J, Pigneux A, Schiller GJ, Jung J, Jane Leonard E, Fingert H, Westervelt P (2014). An exploratory phase 2 study of investigational Aurora A kinase inhibitor alisertib (MLN8237) in acute myelogenous leukemia and myelodysplastic syndromes. Leuk Res Rep.

[70] Bose P, Grant S (2014). Orphan drug designation for pracinostat, volasertib and alvocidib in AML. Leuk Res.

[71] Kaur G, Stetler-Stevenson M, Sebers S, Worland P, Sedlacek H, Myers C, Czech J, Naik R, Sausville E (1992). Growth inhibition with reversible cell cycle arrest of carcinoma cells by flavone L86-8275. J Natl Cancer Inst.

[72] Senderowicz AM, Headlee D, Stinson SF, Lush RM, Kalil N, Villalba L, Hill K, Steinberg SM, Figg WD, Tompkins A, Arbuck SG, Sausville EA (1998). Phase I trial of continuous infusion flavopiridol, a novel cyclin-dependent kinase inhibitor, in patients with refractory neoplasms. J Clin Oncol.

[73] Almenara J, Rosato R, Grant S (2002). Synergistic induction of mitochondrial damage and apoptosis in human leukemia cells by flavopiridol and the histone deacetylase inhibitor suberoylanilide hydroxamic acid (SAHA). Leukemia.

[74] Chen R, Keating MJ, Gandhi V, Plunkett W (2005). Transcription inhibition by flavopiridol: mechanism of chronic lymphocytic leukemia cell death. Blood.

[75] Decker RH, Dai Y, Grant S (2001). The cyclin-dependent kinase inhibitor flavopiridol induces apoptosis in human leukemia cells (U937) through the mitochondrial rather than the receptor-mediated pathway. Cell Death Differ.

[76] Kasper S, Breitenbuecher F, Heidel F, Hoffarth S, Markova B, Schuler M, Fischer T (2012). Targeting MCL-1 sensitizes FLT3-ITD-positive leukemias to cytotoxic therapies. Blood Cancer J.

[77] Rosato RR, Almenara JA, Kolla SS, Maggio SC, Coe S, Giménez MS, Dent P, Grant S (2007). Mechanism and functional role of XIAP and Mcl-1 down-regulation in flavopiridol/vorinostat antileukemic interactions. Mol Cancer Ther.

[78] Bose P, Perkins EB, Honeycut C, Wellons MD, Stefan T, Jacobberger JW, Kontopodis E, Beumer JH, Egorin MJ, Imamura CK, Douglas Figg W, Karp JE, Koc ON (2012). Phase I trial of the combination of flavopiridol and imatinib mesylate in patients with Bcr-Abl+ hematological malignancies. Cancer Chemother Pharmacol.

[79] Holkova B, Kmieciak M, Perkins EB, Bose P, Baz RC, Roodman GD, Stuart RK, Ramakrishnan V, Wan W, Peer CJ, Dawson J, Kang L, Honeycutt C (2014). Phase I trial of bortezomib (PS-341; NSC 681239) and “nonhybrid” (bolus) infusion schedule of alvocidib (flavopiridol; NSC 649890) in patients with recurrent or refractory indolent B-cell neoplasms. Clin Cancer Res.

[80] Holkova B, Perkins EB, Ramakrishnan V, Tombes MB, Shrader E, Talreja N, Wellons MD, Hogan KT, Roodman GD, Coppola D, Kang L, Dawson J, Stuart RK (2011). Phase I trial of bortezomib (PS-341; NSC 681239) and alvocidib (flavopiridol; NSC 649890) in patients with recurrent or refractory B-cell neoplasms. Clin Cancer Res.

[81] Karp JE, Blackford A, Smith BD, Alino K, Seung AH, Bolaños-Meade J, Greer JM, Carraway HE, Gore SD, Jones RJ, Levis MJ, McDevitt MA, Doyle LA, Wright JJ (2010). Clinical activity of sequential flavopiridol, cytosine arabinoside, and mitoxantrone for adults with newly diagnosed, poor-risk acute myelogenous leukemia. Leuk Res.

[82] Karp JE, Passaniti A, Gojo I, Kaufmann S, Bible K, Garimella TS, Greer J, Briel J, Smith BD, Gore SD, Tidwell ML, Ross DD, Wright JJ (2005). Phase I and pharmacokinetic study of flavopiridol followed by 1-beta-D-arabinofuranosylcytosine and mitoxantrone in relapsed and refractory adult acute leukemias. Clin Cancer Res.

[83] Lin TS, Blum KA, Fischer DB, Mitchell SM, Ruppert AS, Porcu P, Kraut EH, Baiocchi RA, Moran ME, Johnson AJ, Schaaf LJ, Grever MR, Byrd JC (2010). Flavopiridol, fludarabine, and rituximab in mantle cell lymphoma and indolent B-cell lymphoproliferative disorders. J Clin Oncol.

[84] Zeidner JF, Foster MC, Blackford AL, Litzow MR, Morris LE, Strickland SA, Lancet JE, Bose P, Levy MY, Tibes R, Gojo I, Gocke CD, Rosner GL (2015). Randomized multicenter phase II study of flavopiridol (alvocidib), cytarabine, and mitoxantrone (FLAM) versus cytarabine/daunorubicin (7+3) in newly diagnosed acute myeloid leukemia. Haematologica.

[85] Zeidner JF, Karp JE (2015). Clinical activity of alvocidib (flavopiridol) in acute myeloid leukemia. Leuk Res.

[86] Karp JE, Garrett-Mayer E, Estey EH, Rudek MA, Smith BD, Greer JM, Drye DM, Mackey K, Dorcy KS, Gore SD, Levis MJ, McDevitt MA, Carraway HE (2012). Randomized phase II study of two schedules of flavopiridol given as timed sequential therapy with cytosine arabinoside and mitoxantrone for adults with newly diagnosed, poor-risk acute myelogenous leukemia. Haematologica.

[87] Karp JE, Smith BD, Levis MJ, Gore SD, Greer J, Hattenburg C, Briel J, Jones RJ, Wright JJ, Colevas AD (2007). Sequential flavopiridol, cytosine arabinoside, and mitoxantrone: a phase II trial in adults with poor-risk acute myelogenous leukemia. Clin Cancer Res.

[88] Hanahan D, Weinberg RA (2011). Hallmarks of cancer: the next generation. Cell.

[89] Carlson BA, Dubay MM, Sausville EA, Brizuela L, Worland PJ (1996). Flavopiridol induces G1 arrest with inhibition of cyclin-dependent kinase (CDK) 2 and CDK4 in human breast carcinoma cells. Cancer Res.

[90] Losiewicz MD, Carlson BA, Kaur G, Sausville EA, Worland PJ (1994). Potent inhibition of CDC2 kinase activity by the flavonoid L86-8275. Biochem Biophys Res Commun.

[91] Worland PJ, Kaur G, Stetler-Stevenson M, Sebers S, Sartor O, Sausville EA (1993). Alteration of the phosphorylation state of p34cdc2 kinase by the flavone L86-8275 in breast carcinoma cells. Correlation with decreased H1 kinase activity. Biochem Pharmacol.

[92] Bartek J, Lukas C, Lukas J (2004). Checking on DNA damage in S phase. Nat Rev Mol Cell Biol.

[93] Bartek J, Lukas J (2001). Pathways governing G1/S transition and their response to DNA damage. FEBS Lett.

[94] Sedlacek HH (2001). Mechanisms of action of flavopiridol. Crit Rev Oncol Hematol.

[95] Malumbres M, Barbacid M (2001). To cycle or not to cycle: a critical decision in cancer. Nat Rev Cancer.

[96] Malumbres M, Barbacid M (2009). Cell cycle, CDKs and cancer: a changing paradigm. Nat Rev Cancer.

[97] Malumbres M (2014). Cyclin-dependent kinases. Genome Biol.

[98] Schwartz GK, Shah MA (2005). Targeting the cell cycle: a new approach to cancer therapy. J Clin Oncol.

[99] Hydbring P, Malumbres M, Sicinski P (2016). Non-canonical functions of cell cycle cyclins and cyclin-dependent kinases. Nat Rev Mol Cell Biol.

[100] Satyanarayana A, Kaldis P (2009). Mammalian cell-cycle regulation: several Cdks, numerous cyclins and diverse compensatory mechanisms. Oncogene.

[101] De Azevedo WF, Mueller-Dieckmann HJ, Schulze-Gahmen U, Worland PJ, Sausville E, Kim SH (1996). Structural basis for specificity and potency of a flavonoid inhibitor of human CDK2, a cell cycle kinase. Proc Natl Acad Sci U S A.

[102] Sedlacek H, Czech J, Naik R, Kaur G, Worland P, Losiewicz M, Parker B, Carlson B, Smith A, Senderowicz A, Sausville E (1996). Flavopiridol (L86 8275; NSC 649890), a new kinase inhibitor for tumor therapy. Int J Oncol.

[103] Radhakrishnan SK, Bhat UG, Halasi M, Gartel AL (2008). P-TEFb inhibitors interfere with activation of p53 by DNA-damaging agents. Oncogene.

[104] Rosato RR, Almenara JA, Cartee L, Betts V, Chellappan SP, Grant S (2002). The cyclin-dependent kinase inhibitor flavopiridol disrupts sodium butyrate-induced p21WAF1/CIP1 expression and maturation while reciprocally potentiating apoptosis in human leukemia cells. Mol Cancer Ther.

[105] Chao SH, Fujinaga K, Marion JE, Taube R, Sausville EA, Senderowicz AM, Peterlin BM, Price DH (2000). Flavopiridol inhibits P-TEFb and blocks HIV-1 replication. J Biol Chem.

[106] Chao SH, Price DH (2001). Flavopiridol inactivates P-TEFb and blocks most RNA polymerase II transcription in vivo. J Biol Chem.

[107] Shiekhattar R, Mermelstein F, Fisher RP, Drapkin R, Dynlacht B, Wessling HC, Morgan DO, Reinberg D (1995). Cdk-activating kinase complex is a component of human transcription factor TFIIH. Nature.

[108] Garriga J, Graña X (2014). CDK9 inhibition strategy defines distinct sets of target genes. BMC Res Notes.

[109] Garriga J, Xie H, Obradovic Z, Graña X (2010). Selective control of gene expression by CDK9 in human cells. J Cell Physiol.

[110] Lü X, Burgan WE, Cerra MA, Chuang EY, Tsai MH, Tofilon PJ, Camphausen K (2004). Transcriptional signature of flavopiridol-induced tumor cell death. Mol Cancer Ther.

[111] Parker BW, Kaur G, Nieves-Neira W, Taimi M, Kohlhagen G, Shimizu T, Losiewicz MD, Pommier Y, Sausville EA, Senderowicz AM (1998). Early induction of apoptosis in hematopoietic cell lines after exposure to flavopiridol. Blood.

[112] Bogenberger JM, Kornblau SM, Pierceall WE, Lena R, Chow D, Shi CX, Mantei J, Ahmann G, Gonzales IM, Choudhary A, Valdez R, Camoriano J, Fauble V (2014). BCL-2 family proteins as 5-Azacytidine-sensitizing targets and determinants of response in myeloid malignancies. Leukemia.

[113] Bose P, Grant S (2013). Mcl-1 as a Therapeutic Target in Acute Myelogenous Leukemia (AML). Leuk Res Rep.

[114] Kim W, Soh KK, Lee YS, Peterson P, Whatcott CJ, Siddiqui-Jain A, Weitman S, Bearss DJ, Warner SL (2016). Abstract 3728: targeting MCL-1 expression, through the inhibition of CDK9 and super enhancer driven transcription, offers multiple opportunities for rational drug combinations. Cancer Res.

[115] Chen S, Dai Y, Pei XY, Myers J, Wang L, Kramer LB, Garnett M, Schwartz DM, Su F, Simmons GL, Richey JD, Larsen DG, Dent P (2012). CDK inhibitors upregulate BH3-only proteins to sensitize human myeloma cells to BH3 mimetic therapies. Cancer Res.

[116] Gojo I, Zhang B, Fenton RG (2002). The cyclin-dependent kinase inhibitor flavopiridol induces apoptosis in multiple myeloma cells through transcriptional repression and down-regulation of Mcl-1. Clin Cancer Res.

[117] Kitada S, Zapata JM, Andreeff M, Reed JC (2000). Protein kinase inhibitors flavopiridol and 7-hydroxy-staurosporine down-regulate antiapoptosis proteins in B-cell chronic lymphocytic leukemia. Blood.

[118] Mihara M, Shintani S, Nakashiro K, Hamakawa H (2003). Flavopiridol, a cyclin dependent kinase (CDK) inhibitor, induces apoptosis by regulating Bcl-x in oral cancer cells. Oral Oncol.

[119] Nelson DM, Joseph B, Hillion J, Segal J, Karp JE, Resar LM (2011). Flavopiridol induces BCL-2 expression and represses oncogenic transcription factors in leukemic blasts from adults with refractory acute myeloid leukemia. Leuk Lymphoma.

[120] Yao Y, Shi J, Zhang Z, Zhang F, Ma R, Zhao Y (2013). The radiation-sensitizing effect of flavopiridol in the esophageal cancer cell line Eca109. Oncol Lett.

[121] Zhou L, Zhang Y, Sampath D, Leverson J, Dai Y, Kmieciak M, Nguyen M, Orlowski RZ, Grant S (2018). Flavopiridol enhances ABT-199 sensitivity in unfavourable-risk multiple myeloma cells in vitro and in vivo. Br J Cancer.

[122] Bright SA, Campiani G, Deininger MW, Lawler M, Williams DC, Zisterer DM (2010). Sequential treatment with flavopiridol synergistically enhances pyrrolo-1,5-benzoxazepine-induced apoptosis in human chronic myeloid leukaemia cells including those resistant to imatinib treatment. Biochem Pharmacol.

[123] Gomez LA, de Las Pozas A, Perez-Stable C (2006). Sequential combination of flavopiridol and docetaxel reduces the levels of X-linked inhibitor of apoptosis and AKT proteins and stimulates apoptosis in human LNCaP prostate cancer cells. Mol Cancer Ther.

[124] Rosato RR, Dai Y, Almenara JA, Maggio SC, Grant S (2004). Potent antileukemic interactions between flavopiridol and TRAIL/Apo2L involve flavopiridol-mediated XIAP downregulation. Leukemia.

[125] Wittmann S, Bali P, Donapaty S, Nimmanapalli R, Guo F, Yamaguchi H, Huang M, Jove R, Wang HG, Bhalla K (2003). Flavopiridol down-regulates antiapoptotic proteins and sensitizes human breast cancer cells to epothilone B-induced apoptosis. Cancer Res.

[126] Yu C, Rahmani M, Dai Y, Conrad D, Krystal G, Dent P, Grant S (2003). The lethal effects of pharmacological cyclin-dependent kinase inhibitors in human leukemia cells proceed through a phosphatidylinositol 3-kinase/Akt-dependent process. Cancer Res.

[127] Buratowski S (2009). Progression through the RNA polymerase II CTD cycle. Mol Cell.

[128] Phatnani HP, Greenleaf AL (2006). Phosphorylation and functions of the RNA polymerase II CTD. Genes Dev.

[129] Egly JM, Coin F (2011). A history of TFIIH: two decades of molecular biology on a pivotal transcription/repair factor. DNA Repair (Amst).

[130] Fisher RP (2005). Secrets of a double agent: CDK7 in cell-cycle control and transcription. J Cell Sci.

[131] Larochelle S, Amat R, Glover-Cutter K, Sansó M, Zhang C, Allen JJ, Shokat KM, Bentley DL, Fisher RP (2012). Cyclin-dependent kinase control of the initiation-to-elongation switch of RNA polymerase II. Nat Struct Mol Biol.

[132] Paparidis NF, Durvale MC, Canduri F (2017). The emerging picture of CDK9/P-TEFb: more than 20 years of advances since PITALRE. Mol Biosyst.

[133] Peng J, Marshall NF, Price DH (1998). Identification of a cyclin subunit required for the function of Drosophila P-TEFb. J Biol Chem.

[134] Peng J, Zhu Y, Milton JT, Price DH (1998). Identification of multiple cyclin subunits of human P-TEFb. Genes Dev.

[135] Berg T (2008). Inhibition of transcription factors with small organic molecules. Curr Opin Chem Biol.

[136] Wang Y, Zhang T, Kwiatkowski N, Abraham BJ, Lee TI, Xie S, Yuzugullu H, Von T, Li H, Lin Z, Stover DG, Lim E, Wang ZC (2015). CDK7-dependent transcriptional addiction in triple-negative breast cancer. Cell.

[137] Chipumuro E, Marco E, Christensen CL, Kwiatkowski N, Zhang T, Hatheway CM, Abraham BJ, Sharma B, Yeung C, Altabef A, Perez-Atayde A, Wong KK, Yuan GC (2014). CDK7 inhibition suppresses super-enhancer-linked oncogenic transcription in MYCN-driven cancer. Cell.

[138] Christensen CL, Kwiatkowski N, Abraham BJ, Carretero J, Al-Shahrour F, Zhang T, Chipumuro E, Herter-Sprie GS, Akbay EA, Altabef A, Zhang J, Shimamura T, Capelletti M (2014). Targeting transcriptional addictions in small cell lung cancer with a covalent CDK7 inhibitor. Cancer Cell.

[139] Kwiatkowski N, Zhang T, Rahl PB, Abraham BJ, Reddy J, Ficarro SB, Dastur A, Amzallag A, Ramaswamy S, Tesar B, Jenkins CE, Hannett NM, McMillin D (2014). Targeting transcription regulation in cancer with a covalent CDK7 inhibitor. Nature.

[140] Mansour MR, Abraham BJ, Anders L, Berezovskaya A, Gutierrez A, Durbin AD, Etchin J, Lawton L, Sallan SE, Silverman LB, Loh ML, Hunger SP, Sanda T (2014). Oncogene regulation. An oncogenic super-enhancer formed through somatic mutation of a noncoding intergenic element. Science.

[141] Blackwood EM, Kadonaga JT (1998). Going the distance: a current view of enhancer action. Science.

[142] Wittkopp PJ, Kalay G (2011). Cis-regulatory elements: molecular mechanisms and evolutionary processes underlying divergence. Nat Rev Genet.

[143] Hnisz D, Abraham BJ, Lee TI, Lau A, Saint-André V, Sigova AA, Hoke HA, Young RA (2013). Super-enhancers in the control of cell identity and disease. Cell.

[144] Parker SC, Stitzel ML, Taylor DL, Orozco JM, Erdos MR, Akiyama JA, van Bueren KL, Chines PS, Narisu N, Black BL, Visel A, Pennacchio LA, Collins FS, NISC Comparative Sequencing Program, National Institutes of Health Intramural Sequencing Center Comparative Sequencing Program Authors, NISC Comparative Sequencing Program Authors (2013). Chromatin stretch enhancer states drive cell-specific gene regulation and harbor human disease risk variants. Proc Natl Acad Sci U S A.

[145] Whyte WA, Orlando DA, Hnisz D, Abraham BJ, Lin CY, Kagey MH, Rahl PB, Lee TI, Young RA (2013). Master transcription factors and mediator establish super-enhancers at key cell identity genes. Cell.

[146] Hnisz D, Schuijers J, Lin CY, Weintraub AS, Abraham BJ, Lee TI, Bradner JE, Young RA (2015). Convergence of developmental and oncogenic signaling pathways at transcriptional super-enhancers. Mol Cell.

[147] Zhou Q, Li T, Price DH (2012). RNA polymerase II elongation control. Annu Rev Biochem.

[148] Green DR, Llambi F (2015). Cell Death Signaling. Cold Spring Harb Perspect Biol.

[149] Wu H, Medeiros LJ, Young KH (2018). Apoptosis signaling and BCL-2 pathways provide opportunities for novel targeted therapeutic strategies in hematologic malignances. Blood Rev.

[150] Tait SW, Green DR (2010). Mitochondria and cell death: outer membrane permeabilization and beyond. Nat Rev Mol Cell Biol.

[151] Chipuk JE, Moldoveanu T, Llambi F, Parsons MJ, Green DR (2010). The BCL-2 family reunion. Mol Cell.

[152] US Food and Drug Administration (2016). FDA approves new drug for chronic lymphocytic leukemia in patients with a specific chromosomal abnormality. https://www.fda.gov/NewsEvents/Newsroom/PressAnnouncements/ucm495253.htm.

[153] Pan R, Hogdal LJ, Benito JM, Bucci D, Han L, Borthakur G, Cortes J, DeAngelo DJ, Debose L, Mu H, Döhner H, Gaidzik VI, Galinsky I (2014). Selective BCL-2 inhibition by ABT-199 causes on-target cell death in acute myeloid leukemia. Cancer Discov.

[154] DiNardo CD, Pollyea DA, Jonas BA, Konopleva M, Pullarkat V, Wei A, Kantarjian HM, Pigneux A, Recher C, Seymour JF, Dunbar M, Xu T, Mabry M (2017). Updated safety and efficacy of venetoclax with decitabine or azacitidine in treatment-naive, elderly patients with acute myeloid leukemia. Blood.

[155] Wei A, Strickland SA, Roboz GJ, Hou JZ, Fiedler W, Lin TL, Walter RB, Enjeti A, Chyla B, Popovic R, Fakouhi K, Shah P, Dunbar M (2017). Phase 1/2 study of venetoclax with low-dose cytarabine in treatment-naive, elderly patients with acute myeloid leukemia unfit for intensive chemotherapy: 1-year outcomes. Blood.

[156] Fischer U, Forster M, Rinaldi A, Risch T, Sungalee S, Warnatz HJ, Bornhauser B, Gombert M, Kratsch C, Stütz AM, Sultan M, Tchinda J, Worth CL (2015). Genomics and drug profiling of fatal TCF3-HLF-positive acute lymphoblastic leukemia identifies recurrent mutation patterns and therapeutic options. Nat Genet.

[157] Khaw SL, Suryani S, Evans K, Richmond J, Robbins A, Kurmasheva RT, Billups CA, Erickson SW, Guo Y, Houghton PJ, Smith MA, Carol H, Roberts AW (2016). Venetoclax responses of pediatric ALL xenografts reveal sensitivity of MLL-rearranged leukemia. Blood.

[158] (2018). Venetoclax (VENCLEXTA) prescribing information.

[159] Beroukhim R, Mermel CH, Porter D, Wei G, Raychaudhuri S, Donovan J, Barretina J, Boehm JS, Dobson J, Urashima M, Mc Henry KT, Pinchback RM, Ligon AH (2010). The landscape of somatic copy-number alteration across human cancers. Nature.

[160] Wei SH, Dong K, Lin F, Wang X, Li B, Shen JJ, Zhang Q, Wang R, Zhang HZ (2008). Inducing apoptosis and enhancing chemosensitivity to gemcitabine via RNA interference targeting Mcl-1 gene in pancreatic carcinoma cell. Cancer Chemother Pharmacol.

[161] Yeh YY, Chen R, Hessler J, Mahoney E, Lehman AM, Heerema NA, Grever MR, Plunkett W, Byrd JC, Johnson AJ (2015). Up-regulation of CDK9 kinase activity and Mcl-1 stability contributes to the acquired resistance to cyclin-dependent kinase inhibitors in leukemia. Oncotarget.

[162] Decker RH, Wang S, Dai Y, Dent P, Grant S (2002). Loss of the Bcl-2 phosphorylation loop domain is required to protect human myeloid leukemia cells from flavopiridol-mediated mitochondrial damage and apoptosis. Cancer Biol Ther.

[163] Whatcott C, Bogenberger J, Kim W, Soh K, Lee YS, Peterson P, Maughan K, Siddiqui-Jain A, Weitman S, Bearss D, Warner S, Tibes R Abstract P557: Alvocidib potentiates the activity of ABT-199 in nonclinical models of Acute Myeloid Leukemia. https://learningcenter.ehaweb.org/eha/2016/21st/133445/clifford.whatcott.alvocidib.potentiates.the.activity.of.abt-199.in.nonclinical.html.

[164] Perciavalle RM, Stewart DP, Koss B, Lynch J, Milasta S, Bathina M, Temirov J, Cleland MM, Pelletier S, Schuetz JD, Youle RJ, Green DR, Opferman JT (2012). Anti-apoptotic MCL-1 localizes to the mitochondrial matrix and couples mitochondrial fusion to respiration. Nat Cell Biol.

[165] Perciavalle RM, Opferman JT (2013). Delving deeper: MCL-1's contributions to normal and cancer biology. Trends Cell Biol.

[166] Adams KW, Cooper GM (2007). Rapid turnover of mcl-1 couples translation to cell survival and apoptosis. J Biol Chem.

[167] Eckelman BP, Salvesen GS, Scott FL (2006). Human inhibitor of apoptosis proteins: why XIAP is the black sheep of the family. EMBO Rep.

[168] Salvesen GS, Duckett CS (2002). IAP proteins: blocking the road to death's door. Nat Rev Mol Cell Biol.

[169] Choi YE, Butterworth M, Malladi S, Duckett CS, Cohen GM, Bratton SB (2009). The E3 ubiquitin ligase cIAP1 binds and ubiquitinates caspase-3 and -7 via unique mechanisms at distinct steps in their processing. J Biol Chem.

[170] Eckelman BP, Salvesen GS (2006). The human anti-apoptotic proteins cIAP1 and cIAP2 bind but do not inhibit caspases. J Biol Chem.

[171] Rahmani M, Davis EM, Bauer C, Dent P, Grant S (2005). Apoptosis induced by the kinase inhibitor BAY 43-9006 in human leukemia cells involves down-regulation of Mcl-1 through inhibition of translation. J Biol Chem.

[172] Belmar J, Fesik SW (2015). Small molecule Mcl-1 inhibitors for the treatment of cancer. Pharmacol Ther.

[173] Richard DJ, Lena R, Bannister T, Blake N, Pierceall WE, Carlson NE, Keller CE, Koenig M, He Y, Minond D, Mishra J, Cameron M, Spicer T (2013). Hydroxyquinoline-derived compounds and analoguing of selective Mcl-1 inhibitors using a functional biomarker. Bioorg Med Chem.

[174] Kotschy A, Szlavik Z, Murray J, Davidson J, Maragno AL, Le Toumelin-Braizat G, Chanrion M, Kelly GL, Gong JN, Moujalled DM, Bruno A, Csekei M, Paczal A (2016). The MCL1 inhibitor S63845 is tolerable and effective in diverse cancer models. Nature.

[175] Caenepeel SR, Belmontes B, Sun J, Coxon A, Moody G, Hughes PE (2017). Abstract 2027: Preclinical evaluation of AMG 176, a novel, potent and selective Mcl-1 inhibitor with robust anti-tumor activity in Mcl-1 dependent cancer models. Cancer Res.

[176] Hird AW, Secrist JP, Adam A, Belmonte MA, Gangl E, Gibbons F, Hargreaves D, Johannes JW, Kazmirski SL, Kettle JG, Kurtz SE, Lamb ML, Packer MJ (2017). Abstract DDT01-02: AZD5991: A potent and selective macrocyclic inhibitor of Mcl-1 for treatment of hematologic cancers. Cancer Res.

